# Association of *interleukin-18* gene polymorphism with body mass index in women

**DOI:** 10.1186/1477-7827-10-31

**Published:** 2012-04-24

**Authors:** Hye-Lin Kim, Sung One Cho, Seon-Young Kim, Sung-Hoon Kim, Won-Seok Chung, Seok-Hee Chung, Sung-Soo Kim, Seong-Gyu Ko, Chang-Hyun Jeong, Su-Jin Kim, Seung-Heon Hong, Jae-Young Um

**Affiliations:** 1 College of Korean Medicine, Institute of Korean Medicine, Kyung Hee University, 1 Heogi-dong, Dongdaemun-gu, Seoul, 130-701, Republic of Korea; 2 Department of Oriental Pharmacy, College of Pharmacy, Wonkwang University, Iksan, Jeonbuk, 570-749, Republic of Korea; 3 Department of Cosmeceutical Science, Daegu Hanny University 290, Yugok-Dong, Kyungsan, 712-715, Republic of Korea

**Keywords:** Obesity, Interleukin-18, Polymorphism, Korean

## Abstract

**Background:**

Interleukin (IL)-18 is an important regulator of innate and acquired immune responses and has multiple roles in chronic inflammation and autoimmune disorders. Obesity is characterized by low- grade chronic inflammation. IL-18 has been suggested as an adipogenic cytokine that is associated with excess adiposity. The purpose of this study is to evaluate the relationship between *IL-18* gene polymorphisms (−137 G/C and −607 C/A) and obesity.

**Methods:**

All 680 subjects were genotyped for the polymorphisms of *IL-18* gene promoters (at positions −137 G/C and −607 C/A) using a polymerase chain reaction (271 cases with BMI ≥25 kg/m^2^ and 409 controls with BMI <25 kg/m^2^). A chi-square test was used to compare the genotype and allele frequencies between the cases and control populations.

**Results:**

Analyses of the genotype distributions revealed that *IL-18* –607 C/A polymorphism was associated with an increase in body mass index in obese women in the Korean population (chi(2) = 12.301, *df* = 2, *p* = 0.015).

**Conclusion:**

Carriage of the A allele at position −607 in the promoter of the *IL-18* gene may have a role in the development of obesity.

## Background

Obesity is increasing rapidly among women all over the world, and more women in fertile ages become overweight and obese [1]. Among all other problems, women who are obese have higher rates of amenorrhoea and infertility [1]. Over the past decade, research has associated obesity with inflammation; this association was first proposed in the seminal article by Hatamilsligil et al. [2]. In this article, tumor necrosis factor-α (TNF-α) was shown to be constitutively expressed via adipose tissue, to be hyperexpressed in obesity, and to mediate insulin resistance in the major animal models of obesity. Furthermore, the neutralization of TNF-α with soluble TNF-α receptors resulted in the restoration of insulin sensitivity [2]. Thus, obesity is now characterized by a state of low- grade inflammation that is associated with the increase of cytokine production. Adipose tissue, which is infiltrated by monocytes and macrophages in obesity, secretes numerous soluble mediators including adipokines such as adiponectin or leptin and many classical cytokines such as TNF-α, interleukin (IL)-6, and IL-1 family members [3,4].

IL-18 has been suggested as an adipogenic cytokine [5] that is associated with excess adiposity [6]. Adipocytes from obese individuals produce higher levels of IL-18 compared with lean individuals and higher circulating IL-18 levels were observed in obese individuals and those with a high body mass index (BMI), insulin resistance, hypertriglyceridemia, and metabolic syndrome [6-9]. Deficiencies of *IL-18* in mice (*Il18*^*−/−*^) led to the exhibition of late- onset obesity and insulin resistance [10]. Therefore, it has been hypothesized that the increased IL-18 concentrations have a pathophysiological role in obesity and metabolic syndromes.

Obesity has a strong genetic etiology involving numerous identified metabolic pathways and others not yet examined. *IL-18* promoter polymorphisms have been associated with various inflammatory diseases, and three single nucleotide polymorphisms (SNPs) in the promoter of the *IL-18* gene at the positions −656 G/T, −607 C/A and −137 G/C have been identified. The functional significance of the two SNPs of the C allele at position −607 and the G allele at position −137 is attributed to the higher transcription and protein production of IL-18 [11,12]. In order to investigate the possible roles of the SNPs from the *IL-18* gene promoter region (−137 G/C and −607 C/A) in the development of obesity, the genetic polymorphisms of obese subjects were evaluated.

## Methods

### Subjects

Subjects were recruited consecutively from an obesity clinic at a Korean hospital; they were placed into an ongoing project that investigates candidate genes for obesity among the Korean population. The present study population included 680 individuals: 271 were chosen as cases (BMI ≥25 kg/m^2^) and 409 were chosen as controls (BMI <25 kg/m^2^). The 271 cases (BMI ≥25 kg/m^2^) consisted of 170 female subjects and 101 male subjects. All cases were nonsmokers and had no evidence of cancer, liver, renal, hematological disease or other metabolic disorders other than obesity. Details of the recruitment, body composition assessment, and biochemical analysis have been described previously [13]. All participants gave informed consent prior to participating in the research, which was approved by the local ethics committee in accordance with the Helsinki Declaration. Some subjects had participated in the research reported previously [13].

### Phenotype measurements

#### Anthropometry

Height (in cm) and weight (in kg) were measured in order to calculate the BMI as weight (kg)/height (m) squared. The waist circumference (measured at the narrowest point above the hip) was divided by the circumference of the hip (measured at its greatest gluteal protuberance) in order to obtain the waist-to-hip ratio (WHR).

#### Dual-energy X-ray absorptiometry

The fat mass was determined using dual energy X-ray absorptiometry.

### Genotyping

Genomic DNA was extracted from peripheral blood samples using the Exgene^TM^ Blood SV Kit (GeneAll, Korea) as per the manufacturer’s instructions. The concentration of DNA was estimated via absorbance at 260 nm. The SNPs were noted at position −137 (G/C) and −607 (C/A) in the promoter region of the *IL-18* gene, located at chromosome 11q22.2-q22.3. For the position −137-specific PCR, a common reverse primer and two sequence- specific forward primers were used to amplify the 261-bp product. A control forward primer was used to amplify the 446-bp fragment covering the polymorphic site as an internal positive amplification control. PCR was performed in a 20 μl volume containing 0.5 μM of one sequence specific primer and −137 R, 0.3 μM of −137 CTRL, 20 mM Tris–HCl (pH 8.4), 50 mM KCl, 1.5 mM MgCl_2_, 200 μM/dNTPs, 1 U of Taq DNA polymerase, and 200 ng genomic DNA. The cycling conditions were 2 min at 94°C, followed by 5 cycles of 20 s at 94°C and 60 s at 67°C and 25 cycles of 20 s at 94°C, 20 s at 61°C and 40 s at 72°C [14].

For the position −607-specific PCR, a common reverse primer and two sequence- specific forward primers were used to amplify the 196-bp product. A control forward primer was used to amplify the 301-bp fragment covering the polymorphic site as an internal positive amplification control. PCR was performed in a 20 μl volume containing 0.4 μM of one sequence specific primer and −607 R, 0.13 μM of −607 CTRL, 20 mM Tris–HCl (pH 8.4), 50 mM KCl, 1.5 mM MgCl_2_, 200 μM/dNTPs, 1 U of Taq DNA polymerase, and 200 ng genomic DNA. The cycling conditions were 2 min at 94°C, followed by 7 cycles of 20 s at 94°C, 40 s at 64°C, and 40 s at 72°C, and 25 cycles of 20 s at 94°C, 40 s at 57°C, and 40 s at 72°C [14]. All PCR products were separated in 2% agarose gels stained with ethidium bromide. The sequence- specific primers for the position −137 and −607- specific PCR are listed on Table 1.

**Table 1:** **Sequence-specific primers for G/C and C/A alleles and their PCR product sizes for positions −137 and −607 in the promoter of the*IL-18*gene**

**Sequence-specific Primers**	**Primer sequence**	**Product size (bp)**
***G/C allele at position*−*137***		
Common reverse primer	5’–AGGAGGGCAAAATGCACTGG–3’	
forward primers 1	5’–CCCCAACTTTTACGGAAGAAAAG–3’	261
forward primers 2	5’–CCCCAACTTTTACGGAAGAAAAC–3’	261
Control forward primer	5’–CCAATAGGACTGATTATTCCGCA–3’	446
***C/A allele at position*−*607***		
Common reverse primer	5’–TAACCTCATTCAGGACTTCC–3’	
forward primers 1	5’–GTTGCAGAAAGTGTAAAAATTATTAC–3’	196
forward primers 2	5’–GTTGCAGAAAGTGTAAAAATTATTAA–3’	196
Control forward primer	5’–CTTTGCTATCATTCCAGGAA–3’	301

### Statistical analysis

A χ^2^ test was used to compare the genotype and allele frequencies between the cases and control populations. All statistical analyses were performed using SPSS v17.00 (SPSS Inc.) statistical analysis software. Hardy-Weinberg equilibrium was tested by chi-squared test and the haplotype analyses were done using HapAnalyzer version 1.0 (http://hap.ngri.go.kr). The associations between allele frequencies of SNPs and cases were estimated by computing the odds ratios and their 95% confidence intervals with logistic regression analyses controlling for age. Genetic Power Calculator (http://pngu.mgh.harvard.edu/~purcell/gpc) [15] was used to compute the statistical power of our sample. A *p*- value of less than 0.05 was considered statistically significant.

## Results

### Clinical characteristics of subjects according to BMI

The clinical characteristics of the cases according to their BMI are listed in Table 2. In order to obtain a better separation between phenotypes, the subjects were divided into three BMI groups: moderately overweight (BMI 25 ~ 26.9 kg/m^2^), severely overweight (BMI 27 ~ 29.9 kg/m^2^), and obese (BMI ≥30 kg/m^2^). As expected, the total cholesterol, triglyceride, fat mass, percentage of body fat (PBF), and WHR increased in proportion to the BMI values.

**Table 2:** Characteristics of obese subjects according to BMI

	**BMI (kg/m**^**2**^**)**			***p***^**a**^
	**25 ~ 26.9**	**27 ~29.9**	**≥ 30**	
Age (year)	28.9 ± 11.2	31.3 ± 10.54	33.1 ± 12.9	.123
Weight (kg)	64.7 ± 4.7	71.3 ± 6.2	84.5 ± 12.9	< 0.001
Height (cm)	158.3 ± 5.9	159.0 ± 5.9	160.8 ± 5.6	.029
Total cholesterol (mg/dL)	184.3 ± 46.0	184.2 ± 36.6	189.0 ± 40.4	.728
Triglyceride (mg/dL)	117.0 ± 112.4	110.3 ±70.7	136.3 ± 112.8	.251
Fat mass (kg)	23.3 ± 2.3	26.6 ± 2.9	36.4 ± 8.1	< 0.001
PBF (%)	36.1 ± 2.7	37.0 ± 5.0	41.9 ± 4.3	< 0.001
WHR	0.89 ± 0.03	0.92 ± 0.03	0.99 ± .07	< 0.001

Values are means ± S.D.

*BMI* = body mass index, *PBF* = percentage of body fat, *WHR* = Waist-to-hip ratio.

^a^By one-way Anova analysis (among BMI groups).

### Allele and haplotype frequencies in obese cases and controls

The genotype and allele frequencies for *IL-18* polymorphisms are summarized in Table 3. The genotype frequencies were in agreement with the Hardy-Weinberg equilibrium. The power of the sample size was calculated to verify our data using a genetic power calculator [15]. The sample powers of the SNPs were more than 95% (α = 0.05, genotype relative risk = 2-fold). For the −137 genotypes from the 271 cases with BMI ≥25 kg/m^2^, 208 had the GG type (76.8%), 56 the GC type (20.7%), and 7 the CC type (2.6%) genotypes. Of the 409 controls with BMI <25 kg/m^2^, 290 had the GG type (70.9%), 110 the GC type (26.9%), and 9 the CC type (2.2%) genotypes. Regarding the −607 genotypes, 69 of the 271 cases with BMI ≥25 kg/m^2^ had the CC type (25.5%), 157 the CA type (57.9%), and 45 the AA type (16.6%) genotypes. 94 of the 409 controls with BMI <25 kg/m^2^ were type CC (23.0%), 231 were CA (56.5%), and 84 were AA (20.5%) genotypes. No significant differences were observed in the genotype distribution or allele frequency between the cases and controls.

**Table 3:** **Genotype frequencies of*IL-18*gene promoter polymorphism of 271 cases and 409 controls**

	**Cases (BMI ≥25), n (%)**	**Controls (BMI <25), n (%)**	**χ**^**2**^	***p***^***a***^
Position −137 genotype GG	208 (76.8)	290 (70.9)	3.455	.178
GC	56 (20.7)	110 (26.9)		
CC	7 (2.6)	9 (2.2)		
**Alleles**				
G	472 (87.1)	690 (84.4)	1.957	.162
C	70 (12.9)	128 (15.6)		
Position −607 genotype	69 (25.5)	94 (23.0)		
CC				
CA	157 (57.9)	231 (56.5)	1.807	.405
AA	45 (16.6)	84 (20.5)		
Alleles				
C	295 (54.4)	419 (51.2)	1.343	.246
A	247 (45.6)	399 (48.8)		

^a^ By χ^2^-test (2-sided).

The haplotype frequencies were estimated based on genotypes of the *IL-18* polymorphisms. The four haplotyes (haplotype I, II, III, and IV) of the *IL-18* promoter at positions −607 and −137 were present in both cases (BMI ≥25 kg/m^2^) and controls (BMI <25 kg/m^2^) and are presented in Table 4. The frequencies of haplotypes I, II, III, and IV in the cases were 55.5%, 0%, 33.6%, and 10.9%, respectively. The frequencies of haplotypes I, II, III, and IV in the controls were 50.4%, 0.6%, 34.7%, and 14.3%, respectively. No significant differences were observed in the haplotype frequencies between the cases and the controls.

**Table 4:** **Haplotype frequencies of*IL-18*gene promoter polymorphism in case alleles and control alleles**

**Haplotype**	**−607 C/A**	**−137 G/C**	**Cases (BMI ≥25), n (%)**	**Controls (BMI <25), n (%)**	**χ**^**2**^	***p***^**a**^
I	C	G	301 (55.5)	412 (50.4)	3.491	.062
II	C	C	0 (0)	5 (0.6)	3.325	.068
III	A	G	182 (33.6)	284 (34.7)	0.188	.665
IV	A	C	59 (10.9)	117 (14.3)	3.380	.066
Total			542	818		

^a^ By χ^2^-test (2-sided).

### Relationship between *IL-18* polymorphism and BMI seen in obese women

In order to further evaluate the association between the *IL-18* polymorphisms and BMI, only female subjects, 170 of the 271 cases with BMI ≥25 kg/m^2^, were grouped according to their BMI range. The distributions of the GG, GC, and CC genotypes of position −137 and the distributions of the CC, CA, and AA genotypes of position −607 in these cases are shown in Table 5. A significant difference was found in the −607 C/A genotype distribution among the BMI subgroups (χ^2^ = 12.301, *df* = 2, *p* = 0.015). Further analysis showed that the frequency of position −607 AA genotype was higher in a subgroup with BMI ≥30 kg/m^2^ than in both moderately overweight (BMI 25 ~ 26.9 kg/m^2^) and severely overweight (BMI 27 ~ 29.9 kg/m^2^) groups (χ^2^ = 7.729, *df* = 2, *p* = 0.021 and χ^2^ = 8.182, *df* = 2, *p* = 0.017, respectively). However, a difference in the distributions of the position −137 between BMI subgroups was not observed in the obese women.

**Table 5:** **Frequencies of*IL-18*gene promoter polymorphism according to BMI in female cases with BMI ≥25 kg/m**^**2**^**(n = 170)**

	**BMI (kg/m**^**2**^**)**			**χ**^**2**^	***p***^***a***^
	**25 ~26.9, n (%)**	**27 ~ 29.9, n (%)**	**≥ 30, n (%)**		
Position −137	34 (75.6)	52 (76.5)	44 (77.2)	1.212	.876
genotype					
GG					
GC	9 (20.0)	15 (22.1)	12 (21.1)		
CC	2 (4.4)	1 (1.5)	1 (1.8)		
Position −607	12 (32.4)	20 (27.4)	10 (16.7)		
genotype					
CC					
CA	22 (59.5)	45 (61.6)	32 (53.3)	12.301	.015
AA	3 (8.1)	8 (11.0)	18 (30.0)^*,†^		

^a^ By χ^2^-test (2-sided), among three BMI subgroups.

^*^ By χ^2^-test (2-sided), *p* = 0.021, BMI 25 ~ 26.9 kg/m^2^ group versus BMI ≥30 kg/m^2^ group.

^**†**^ By χ^2^-test (2-sided), *p* = 0.017, BMI 27 ~ 29.9 kg/m^2^ group versus BMI ≥30 kg/m^2^ group.

## Discussion

This study determined whether the promoter polymorphisms of *IL-18* gene were associated with obesity and anthropometric parameters in obese women. Obesity is a complex metabolic disorder with a strong genetic component [16]. There are many candidate genes for obesity and its related phenotypes [16]. Most of these genes are candidates for obesity because their mutations cause rare genetic syndromes that affect the adipocyte differentiation [17]. However, the association between inflammatory cytokine genes and obesity has been studied less frequently when compared with other candidate genes. Thus, the association between the polymorphism of *IL-18*, a member of the *IL-1* family, and obesity without metabolic disease was a primary focus of this study.

In this study, an association between the polymorphism in the *IL-18* gene and BMI in women was found. The frequency of haplotype I, which has the C allele at position −607 and the G allele at position −137, was higher in the subjects with BMI ≥25 kg/m^2^ than in the control subjects with BMI <25 kg/m^2^, although the statistical significance was marginal. In addition, there was an apparent association between the −607 C/A polymorphism in *IL-18* and obesity in women.

The functional significance of the two SNPs of the C allele at position −607 and the G allele at position −137 is attributed to the higher transcription and protein production of IL-18 [11,12]. From the results presented here, it was observed that the frequency of haplotype I (the C allele at position −607 and the G allele at position −137) in the cases with BMI ≥25 kg/m^2^ was higher than that in the controls with BMI <25 kg/m^2^. Therefore, it can be inferred that the mechanism by which the *IL-18* gene polymorphism might influence obesity is related to different IL-18 synthesis, secretion, and activity. Indeed, several studies have shown elevated circulating IL-18 concentrations in subjects with obesity and insulin resistance [6,18-20]. In addition, serum IL-18 was increased in obese women, and it declined as body weight was lost [21].

The human *IL-18* gene is located on chromosome 11q22.2-q23.3. Three SNPs in the promoter of the *IL-18* gene at positions −656 G/T, −607 C/A and −137 G/C have been identified. These promoter SNPs have been implicated as susceptibility loci for various diseases, including asthma [22], pulmonary tuberculosis [23], inflammatory bowel disease [24], Parkinson’s disease [25], polycystic ovary syndrome [26], type I diabetes [27], and allergic disorders [28].

It now appears that obesity is associated with a low- grade inflammation of the white adipose tissue resulting from the chronic activation of the innate immune system as the IL-1 family. Until recently, there were four members of the IL-1 family: IL-1α, IL-1β, IL-1 receptor antagonist (IL-1ra), and IL-18 [29]. Previous studies have described an association between the *IL-1* family gene polymorphism and obesity. Manica-Cattani et al. [30] and Lee et al. [16] reported that *IL-1β* polymorphism (+3953 C/T) is linked to the development of obesity. Song et al. [31] also suggested that *IL-1α* polymorphism (−889 C/T) is associated with obesity in women. In addition, Strandberg et al. [32,33] demonstrated that the *IL-1* system gene polymorphisms are associated with fat mass in men. The present study is the first approach in exploring the role of the *IL-18* gene promoter polymorphism in the etiology of obesity in the Korean population. In addition, obesity is increasing rapidly among women all over the world. Obese women have a higher risk than nonobese women of infertility and pregnancy. The loss of as little as 5% of body weight is accompanied by an increase in ovulation rates and reduces biochemical abnormalities [34]. Therefore, the present study might allow targeted therapies to be developed to improve reproductive health in obese women.

## Conclusions

In the present study, two polymorphisms in the promoter regions of the *IL-18* gene were identified. An association between the −607 C/A polymorphism and BMI were demonstrated in women. The results suggest that the −607 C/A polymorphism of the *IL-18* gene may have a role in the development of obesity.

## Competing interests

The authors have declared that no conflict of interest exists.

## Authors’ contributions

Conceived and designed the experiments: JYU and SHH. Performed the experiments: HLK, SOC, SYK, and SJK. Analyzed and interpreted the data: JYU, SHK, WSC, SHC, SSK, CHJ, and SGK. Contributed Wrote the paper: HLK, SOC, and JYU. All authors read and authorized the final manuscript.
